# 4-Di­phenyl­phosphanyl-8-methyl-1,5-naphthyridine

**DOI:** 10.1107/S1600536813013196

**Published:** 2013-05-18

**Authors:** Chen Chen, Kun-Yan Wang, Jin-Fang Liu, Dan-Feng Wang, Hong-Jun Zhu

**Affiliations:** a Department of Applied Chemistry, College of Science, Nanjing University of Technology, Nanjing 210009, People’s Republic of China

## Abstract

In the title compound, C_21_H_17_N_2_P, the dihedral angles between the 1,5-naphthyridine ring system (r.m.s. deviation = 0.005 Å) and the phenyl rings are 89.18 (8) and 77.39 (8)°. The phenyl rings are almost perpendicular, making a dihedral angle of 88.12 (8)°. The only possible inter­molecular inter­action is a very weak aromatic π–π stacking inter­action [centroid–centroid separation = 3.898 (2) Å].

## Related literature

For further synthetic details and background to the role of the title compound as an inter­mediate in the synthesis of OLED materials, see: Chen *et al.* (2012 ▶).
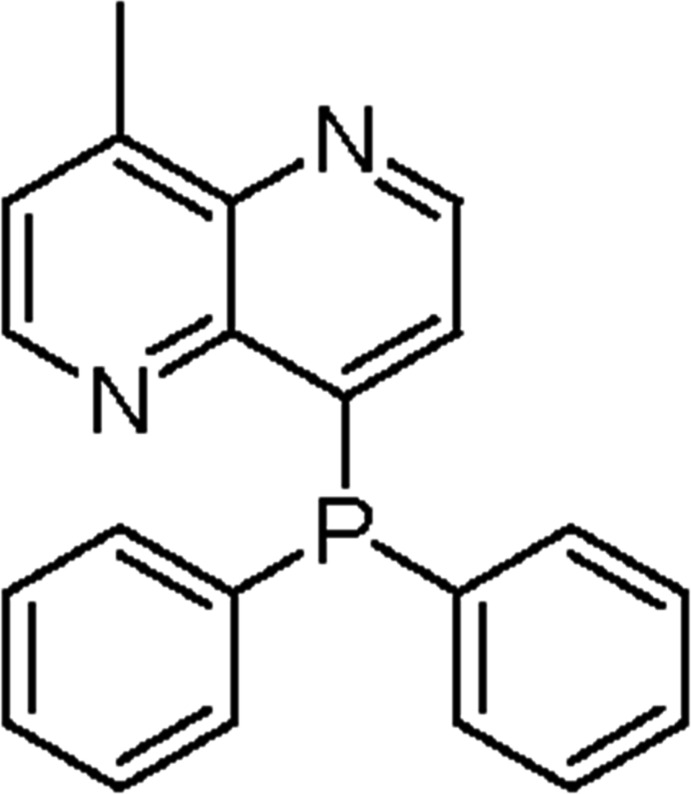


## Experimental

### 

#### Crystal data

C_21_H_17_N_2_P
*M* *_r_* = 328.34
Triclinic, 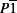

*a* = 7.2320 (14) Å
*b* = 7.4470 (15) Å
*c* = 16.780 (3) Å
α = 99.78 (3)°
β = 93.35 (3)°
γ = 98.58 (3)°
*V* = 877.4 (3) Å^3^
*Z* = 2
Mo *K*α radiation
μ = 0.16 mm^−1^
*T* = 293 K
0.30 × 0.20 × 0.10 mm

#### Data collection

Enraf–Nonius CAD-4 diffractometer
Absorption correction: ψ scan (North *et al.*, 1968 ▶) *T*_min_ = 0.954, *T*_max_ = 0.984
3500 measured reflections
3224 independent reflections
2285 reflections with *I* > 2σ(*I*)
*R*_int_ = 0.023
3 standard reflections every 200 reflections intensity decay: 1%

#### Refinement

*R*[*F* ^2^ > 2σ(*F* ^2^)] = 0.050
*wR*(*F* ^2^) = 0.155
*S* = 1.00
3224 reflections
217 parameters
H-atom parameters constrained
Δρ_max_ = 0.17 e Å^−3^
Δρ_min_ = −0.23 e Å^−3^

### 

Data collection: *CAD-4 EXPRESS* (Enraf–Nonius, 1994 ▶); cell refinement: *CAD-4 EXPRESS*; data reduction: *XCAD4* (Harms & Wocadlo, 1995 ▶); program(s) used to solve structure: *SHELXS97* (Sheldrick, 2008 ▶); program(s) used to refine structure: *SHELXL97* (Sheldrick, 2008 ▶); molecular graphics: *SHELXTL* (Sheldrick, 2008 ▶); software used to prepare material for publication: *SHELXTL*.

## Supplementary Material

Crystal structure: contains datablock(s) I, global. DOI: 10.1107/S1600536813013196/hb7082sup1.cif

Structure factors: contains datablock(s) I. DOI: 10.1107/S1600536813013196/hb7082Isup2.hkl

Supplementary material file. DOI: 10.1107/S1600536813013196/hb7082Isup3.cml

Additional supplementary materials:  crystallographic information; 3D view; checkCIF report

## Figures and Tables

**Fig. 1.**
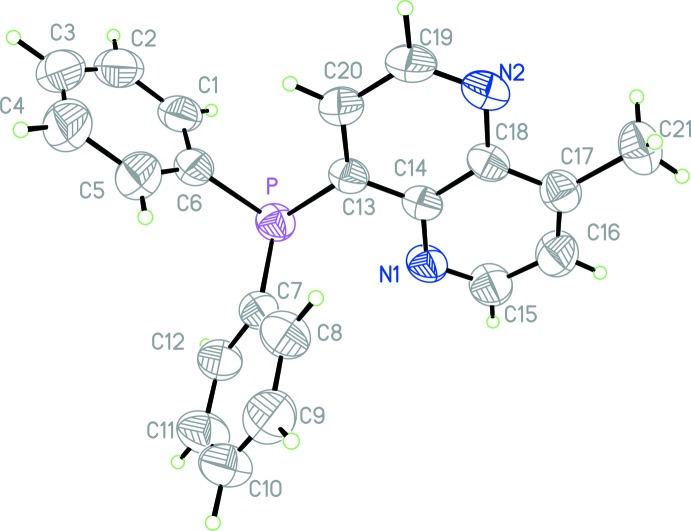
The molecular structure of (I), with displacement ellipsoids drawn at the 50% probability level.

**Fig. 2.**
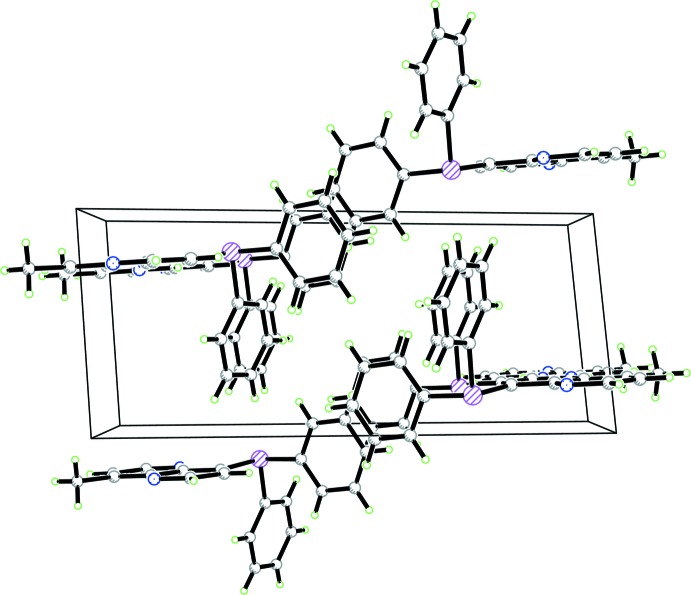
A packing diagram of (I).

## supplementary crystallographic information

### Comment

The tittle compound, (I), is an intermediate for manufacturing OLED materials (Chen *et al.*, 2012). We now report its crystal structure (Fig. 1).

The 1,5-naphthyridine ring system is nearly planar with an r.m.s. deviation of 0.005Å; its mean plane is oriented with respect to the two phenyl rings at 89.18 (8) and 77.39 (8)°. The two phenyl rings are twisted to each other with a dihedral angle of 88.12 (8)°. The crystal packing of the molecules in the crystal is influenced by van der Waals forces (Fig. 2).

### Experimental

The title compund was synthesized according to the published procedure (Chen *et al.*, 2012). Yellow blocks were obtained by dissolving it (0.5 g) in tetrahydrofuran (20 ml) and evaporating the solvent slowly at room temperature for about 5 d.

### Refinement

H atoms were positioned geometrically and refined as riding groups, with C—H = 0.93 Å for aromatic H, and constrained to ride on their parent atoms, with *U*_iso_(H) = *xU*_eq_(C), where *x* = 1.2 for aromatic H, and *x* = 1.5 for other H.

### Crystal data

**Table:**

C_21_H_17_N_2_P	*Z* = 2
*M**_r_* = 328.34	*F*(000) = 344
Triclinic, *P*1	*D*_x_ = 1.243 Mg m^−^^3^
Hall symbol: -P 1	Mo *K*α radiation, λ = 0.71073 Å
*a* = 7.2320 (14) Å	Cell parameters from 25 reflections
*b* = 7.4470 (15) Å	θ = 10–14°
*c* = 16.780 (3) Å	µ = 0.16 mm^−^^1^
α = 99.78 (3)°	*T* = 293 K
β = 93.35 (3)°	Block, yellow
γ = 98.58 (3)°	0.30 × 0.20 × 0.10 mm
*V* = 877.4 (3) Å^3^	

### Data collection

**Table:**

Enraf–Nonius CAD-4 diffractometer	2285 reflections with *I* > 2σ(*I*)
Radiation source: fine-focus sealed tube	*R*_int_ = 0.023
Graphite monochromator	θ_max_ = 25.4°, θ_min_ = 1.2°
ω/2θ scans	*h* = 0→8
Absorption correction: ψ scan (North *et al.*, 1968)	*k* = −8→8
*T*_min_ = 0.954, *T*_max_ = 0.984	*l* = −20→20
3500 measured reflections	3 standard reflections every 200 reflections
3224 independent reflections	intensity decay: 1%

### Refinement

**Table:**

Refinement on *F*^2^	Primary atom site location: structure-invariant direct methods
Least-squares matrix: full	Secondary atom site location: difference Fourier map
*R*[*F*^2^ > 2σ(*F*^2^)] = 0.050	Hydrogen site location: inferred from neighbouring sites
*wR*(*F*^2^) = 0.155	H-atom parameters constrained
*S* = 1.00	*w* = 1/[σ^2^(*F*_o_^2^) + (0.096*P*)^2^] where *P* = (*F*_o_^2^ + 2*F*_c_^2^)/3
3224 reflections	(Δ/σ)_max_ = 0.001
217 parameters	Δρ_max_ = 0.17 e Å^−^^3^
0 restraints	Δρ_min_ = −0.23 e Å^−^^3^

### Special details

**Table:**

Geometry. All e.s.d.'s (except the e.s.d. in the dihedral angle between two l.s. planes) are estimated using the full covariance matrix. The cell e.s.d.'s are taken into account individually in the estimation of e.s.d.'s in distances, angles and torsion angles; correlations between e.s.d.'s in cell parameters are only used when they are defined by crystal symmetry. An approximate (isotropic) treatment of cell e.s.d.'s is used for estimating e.s.d.'s involving l.s. planes.
Refinement. Refinement of *F*^2^ against ALL reflections. The weighted *R*-factor *wR* and goodness of fit *S* are based on *F*^2^, conventional *R*-factors *R* are based on *F*, with *F* set to zero for negative *F*^2^. The threshold expression of *F*^2^ > σ(*F*^2^) is used only for calculating *R*-factors(gt) *etc*. and is not relevant to the choice of reflections for refinement. *R*-factors based on *F*^2^ are statistically about twice as large as those based on *F*, and *R*- factors based on ALL data will be even larger.

### Fractional atomic coordinates and isotropic or equivalent isotropic displacement parameters (Å^2^)

**Table:**

	*x*	*y*	*z*	*U*_iso_*/*U*_eq_	
P	0.18105 (10)	0.17196 (9)	0.71974 (4)	0.0529 (2)	
N1	0.2284 (3)	0.3164 (3)	0.89550 (13)	0.0603 (6)	
C1	−0.0068 (5)	−0.0965 (4)	0.59708 (17)	0.0711 (8)	
H1B	−0.1116	−0.0650	0.6223	0.085*	
N2	0.2453 (3)	−0.1579 (3)	0.92992 (13)	0.0569 (6)	
C2	−0.0308 (6)	−0.2312 (5)	0.5287 (2)	0.0906 (11)	
H2B	−0.1507	−0.2917	0.5087	0.109*	
C3	0.1213 (7)	−0.2762 (5)	0.4901 (2)	0.0976 (13)	
H3A	0.1048	−0.3655	0.4432	0.117*	
C4	0.2974 (6)	−0.1907 (5)	0.5202 (2)	0.0914 (11)	
H4A	0.4009	−0.2230	0.4942	0.110*	
C5	0.3227 (5)	−0.0553 (4)	0.58980 (18)	0.0734 (8)	
H5A	0.4433	0.0028	0.6098	0.088*	
C6	0.1710 (4)	−0.0060 (3)	0.62951 (15)	0.0579 (7)	
C7	0.4103 (3)	0.3111 (3)	0.71826 (14)	0.0501 (6)	
C8	0.5758 (4)	0.2855 (4)	0.75679 (17)	0.0644 (7)	
H8A	0.5754	0.1889	0.7854	0.077*	
C9	0.7416 (4)	0.4013 (4)	0.75339 (19)	0.0757 (8)	
H9A	0.8516	0.3842	0.7806	0.091*	
C10	0.7445 (5)	0.5412 (4)	0.7101 (2)	0.0822 (10)	
H10A	0.8568	0.6181	0.7071	0.099*	
C11	0.5835 (5)	0.5682 (4)	0.6713 (2)	0.0844 (10)	
H11A	0.5859	0.6635	0.6419	0.101*	
C12	0.4175 (4)	0.4557 (3)	0.67539 (17)	0.0652 (7)	
H12A	0.3080	0.4764	0.6491	0.078*	
C13	0.2210 (3)	0.0390 (3)	0.79998 (14)	0.0476 (6)	
C14	0.2313 (3)	0.1306 (3)	0.88168 (14)	0.0468 (6)	
C15	0.2393 (5)	0.3962 (4)	0.97172 (18)	0.0744 (9)	
H15A	0.2379	0.5226	0.9827	0.089*	
C16	0.2527 (4)	0.3065 (4)	1.03778 (18)	0.0736 (8)	
H16A	0.2609	0.3736	1.0903	0.088*	
C17	0.2539 (3)	0.1222 (4)	1.02590 (15)	0.0557 (6)	
C18	0.2437 (3)	0.0284 (3)	0.94493 (15)	0.0478 (6)	
C19	0.2366 (4)	−0.2382 (3)	0.85361 (17)	0.0638 (7)	
H19A	0.2389	−0.3645	0.8426	0.077*	
C20	0.2241 (4)	−0.1471 (3)	0.78760 (16)	0.0576 (6)	
H20A	0.2179	−0.2133	0.7350	0.069*	
C21	0.2657 (4)	0.0179 (4)	1.09549 (16)	0.0724 (8)	
H21A	0.2718	0.1022	1.1460	0.109*	
H21B	0.3761	−0.0394	1.0935	0.109*	
H21C	0.1565	−0.0752	1.0911	0.109*	

### Atomic displacement parameters (Å^2^)

**Table:**

	*U*^11^	*U*^22^	*U*^33^	*U*^12^	*U*^13^	*U*^23^
P	0.0594 (4)	0.0457 (4)	0.0574 (4)	0.0135 (3)	0.0070 (3)	0.0149 (3)
N1	0.0850 (16)	0.0364 (11)	0.0630 (14)	0.0140 (10)	0.0175 (12)	0.0113 (10)
C1	0.086 (2)	0.0568 (16)	0.0678 (18)	−0.0071 (15)	−0.0020 (16)	0.0218 (14)
N2	0.0624 (14)	0.0436 (11)	0.0676 (14)	0.0070 (10)	0.0070 (11)	0.0191 (10)
C2	0.124 (3)	0.066 (2)	0.070 (2)	−0.022 (2)	−0.013 (2)	0.0194 (17)
C3	0.166 (4)	0.0558 (19)	0.061 (2)	−0.009 (2)	0.003 (2)	0.0081 (15)
C4	0.136 (3)	0.065 (2)	0.077 (2)	0.024 (2)	0.029 (2)	0.0121 (17)
C5	0.091 (2)	0.0604 (17)	0.0681 (18)	0.0135 (15)	0.0148 (16)	0.0055 (14)
C6	0.0759 (18)	0.0475 (14)	0.0505 (14)	0.0035 (13)	0.0023 (13)	0.0162 (11)
C7	0.0615 (15)	0.0364 (12)	0.0539 (14)	0.0121 (11)	0.0124 (12)	0.0061 (10)
C8	0.0623 (17)	0.0624 (17)	0.0735 (18)	0.0144 (14)	0.0100 (14)	0.0214 (14)
C9	0.0608 (18)	0.087 (2)	0.076 (2)	0.0133 (16)	0.0077 (15)	0.0024 (17)
C10	0.082 (2)	0.069 (2)	0.088 (2)	−0.0125 (17)	0.0211 (19)	0.0113 (17)
C11	0.100 (3)	0.0589 (18)	0.096 (2)	−0.0019 (17)	0.017 (2)	0.0296 (17)
C12	0.0796 (19)	0.0476 (14)	0.0709 (18)	0.0105 (14)	0.0071 (14)	0.0175 (13)
C13	0.0499 (14)	0.0399 (12)	0.0547 (14)	0.0068 (10)	0.0084 (11)	0.0121 (10)
C14	0.0460 (13)	0.0402 (12)	0.0560 (14)	0.0065 (10)	0.0102 (11)	0.0121 (10)
C15	0.113 (3)	0.0438 (15)	0.0681 (18)	0.0187 (15)	0.0194 (17)	0.0057 (13)
C16	0.098 (2)	0.0628 (18)	0.0591 (17)	0.0169 (16)	0.0137 (16)	0.0018 (14)
C17	0.0519 (15)	0.0614 (16)	0.0563 (15)	0.0096 (12)	0.0090 (12)	0.0152 (12)
C18	0.0437 (13)	0.0456 (13)	0.0576 (14)	0.0082 (10)	0.0116 (11)	0.0158 (11)
C19	0.082 (2)	0.0361 (13)	0.0733 (18)	0.0077 (12)	0.0043 (15)	0.0130 (12)
C20	0.0713 (17)	0.0409 (13)	0.0591 (15)	0.0070 (12)	0.0045 (13)	0.0072 (11)
C21	0.0725 (19)	0.090 (2)	0.0609 (17)	0.0143 (16)	0.0112 (14)	0.0279 (15)

### Geometric parameters (Å, º)

**Table:**

P—C7	1.823 (3)	C9—C10	1.367 (4)
P—C6	1.825 (3)	C9—H9A	0.9300
P—C13	1.836 (2)	C10—C11	1.358 (5)
N1—C15	1.308 (3)	C10—H10A	0.9300
N1—C14	1.367 (3)	C11—C12	1.369 (4)
C1—C2	1.374 (4)	C11—H11A	0.9300
C1—C6	1.392 (4)	C12—H12A	0.9300
C1—H1B	0.9300	C13—C20	1.370 (3)
N2—C19	1.312 (3)	C13—C14	1.416 (3)
N2—C18	1.369 (3)	C14—C18	1.413 (3)
C2—C3	1.364 (5)	C15—C16	1.394 (4)
C2—H2B	0.9300	C15—H15A	0.9300
C3—C4	1.362 (5)	C16—C17	1.355 (4)
C3—H3A	0.9300	C16—H16A	0.9300
C4—C5	1.391 (4)	C17—C18	1.411 (3)
C4—H4A	0.9300	C17—C21	1.513 (3)
C5—C6	1.380 (4)	C19—C20	1.399 (3)
C5—H5A	0.9300	C19—H19A	0.9300
C7—C8	1.381 (4)	C20—H20A	0.9300
C7—C12	1.390 (3)	C21—H21A	0.9600
C8—C9	1.379 (4)	C21—H21B	0.9600
C8—H8A	0.9300	C21—H21C	0.9600
			
C7—P—C6	102.31 (12)	C10—C11—H11A	119.8
C7—P—C13	102.97 (11)	C12—C11—H11A	119.8
C6—P—C13	100.69 (11)	C11—C12—C7	121.0 (3)
C15—N1—C14	115.8 (2)	C11—C12—H12A	119.5
C2—C1—C6	121.5 (3)	C7—C12—H12A	119.5
C2—C1—H1B	119.3	C20—C13—C14	116.6 (2)
C6—C1—H1B	119.3	C20—C13—P	125.1 (2)
C19—N2—C18	116.9 (2)	C14—C13—P	117.97 (16)
C3—C2—C1	119.9 (3)	N1—C14—C18	122.9 (2)
C3—C2—H2B	120.0	N1—C14—C13	117.7 (2)
C1—C2—H2B	120.0	C18—C14—C13	119.5 (2)
C4—C3—C2	120.2 (3)	N1—C15—C16	125.1 (2)
C4—C3—H3A	119.9	N1—C15—H15A	117.4
C2—C3—H3A	119.9	C16—C15—H15A	117.4
C3—C4—C5	120.1 (4)	C17—C16—C15	120.3 (3)
C3—C4—H4A	119.9	C17—C16—H16A	119.8
C5—C4—H4A	119.9	C15—C16—H16A	119.8
C6—C5—C4	120.8 (3)	C16—C17—C18	117.2 (2)
C6—C5—H5A	119.6	C16—C17—C21	122.4 (3)
C4—C5—H5A	119.6	C18—C17—C21	120.4 (2)
C5—C6—C1	117.5 (3)	N2—C18—C17	119.3 (2)
C5—C6—P	125.9 (2)	N2—C18—C14	122.1 (2)
C1—C6—P	116.6 (2)	C17—C18—C14	118.6 (2)
C8—C7—C12	117.7 (2)	N2—C19—C20	124.6 (2)
C8—C7—P	125.38 (19)	N2—C19—H19A	117.7
C12—C7—P	116.9 (2)	C20—C19—H19A	117.7
C9—C8—C7	120.8 (3)	C13—C20—C19	120.4 (2)
C9—C8—H8A	119.6	C13—C20—H20A	119.8
C7—C8—H8A	119.6	C19—C20—H20A	119.8
C10—C9—C8	120.1 (3)	C17—C21—H21A	109.5
C10—C9—H9A	120.0	C17—C21—H21B	109.5
C8—C9—H9A	120.0	H21A—C21—H21B	109.5
C11—C10—C9	120.0 (3)	C17—C21—H21C	109.5
C11—C10—H10A	120.0	H21A—C21—H21C	109.5
C9—C10—H10A	120.0	H21B—C21—H21C	109.5
C10—C11—C12	120.4 (3)		
			
C6—C1—C2—C3	−1.3 (5)	C7—P—C13—C14	76.6 (2)
C1—C2—C3—C4	1.4 (5)	C6—P—C13—C14	−178.00 (19)
C2—C3—C4—C5	−0.9 (5)	C15—N1—C14—C18	0.5 (4)
C3—C4—C5—C6	0.3 (5)	C15—N1—C14—C13	−179.6 (2)
C4—C5—C6—C1	−0.2 (4)	C20—C13—C14—N1	−180.0 (2)
C4—C5—C6—P	−178.5 (2)	P—C13—C14—N1	−6.0 (3)
C2—C1—C6—C5	0.7 (4)	C20—C13—C14—C18	−0.1 (3)
C2—C1—C6—P	179.2 (2)	P—C13—C14—C18	173.91 (17)
C7—P—C6—C5	19.7 (3)	C14—N1—C15—C16	−0.2 (5)
C13—P—C6—C5	−86.2 (2)	N1—C15—C16—C17	−0.5 (5)
C7—P—C6—C1	−158.59 (19)	C15—C16—C17—C18	0.9 (4)
C13—P—C6—C1	95.5 (2)	C15—C16—C17—C21	−179.2 (3)
C6—P—C7—C8	−91.8 (2)	C19—N2—C18—C17	−179.6 (2)
C13—P—C7—C8	12.4 (2)	C19—N2—C18—C14	0.7 (3)
C6—P—C7—C12	88.9 (2)	C16—C17—C18—N2	179.7 (2)
C13—P—C7—C12	−166.89 (19)	C21—C17—C18—N2	−0.2 (3)
C12—C7—C8—C9	0.7 (4)	C16—C17—C18—C14	−0.6 (4)
P—C7—C8—C9	−178.5 (2)	C21—C17—C18—C14	179.5 (2)
C7—C8—C9—C10	−1.4 (4)	N1—C14—C18—N2	179.6 (2)
C8—C9—C10—C11	1.0 (5)	C13—C14—C18—N2	−0.3 (3)
C9—C10—C11—C12	0.0 (5)	N1—C14—C18—C17	−0.1 (4)
C10—C11—C12—C7	−0.7 (5)	C13—C14—C18—C17	180.0 (2)
C8—C7—C12—C11	0.3 (4)	C18—N2—C19—C20	−0.7 (4)
P—C7—C12—C11	179.6 (2)	C14—C13—C20—C19	0.1 (4)
C7—P—C13—C20	−110.0 (2)	P—C13—C20—C19	−173.4 (2)
C6—P—C13—C20	−4.6 (3)	N2—C19—C20—C13	0.3 (4)
